# Trajectories of maternal symptoms of anxiety and depression. A 13-year longitudinal study of a population-based sample

**DOI:** 10.1186/1471-2458-10-589

**Published:** 2010-10-06

**Authors:** Anni Skipstein, Harald Janson, Mike Stoolmiller, Kristin S Mathiesen

**Affiliations:** 1 Norwegian Institute of Public Health, Division of Mental Health, PO Box 4404 Nydalen, 0403 Oslo, Norway; 2 The Norwegian Center for Child Behavioral Development, PO Box 7053 Majorstuen, 0306 Oslo, Norway; 3 Center for Teaching and Learning, University of Oregon, College of Education, Eugene, OR, 97403, USA

## Abstract

**Background:**

There is a lack of population-based studies of developmental trajectories following mothers throughout the whole child-rearing phase and there are few longitudinal studies focusing on both symptoms of depression and anxiety. The aim of the current study is to identify latent trajectory groups based on counts of symptoms of anxiety and depression among mothers throughout the child-rearing phase and the relations of the latent groups to maternal socio-demographic variables.

**Methods:**

Data is from a prospective, longitudinal study of nearly 1000 families in Norway followed from when the index children were 18 months until they were 14.5 years old (the TOPP study). The study used latent profile analysis (LPA) to identify latent groups of mothers with distinct trajectories across time of symptom counts. Latent group differences on socio-demographic variables were tested with one-way ANOVAs, chi-square tests and exact tests.

**Results:**

Six trajectories based on maternal scores from six waves of data collection of symptoms of anxiety and depression were identified; a 'No symptoms' group with mothers without symptoms; a 'Low' group with mothers reporting low symptom levels; a 'Moderate-low' group with mothers reporting moderately low symptom levels; a 'Moderate' group with mothers with moderate symptoms; a 'High-chronic' group with mothers with overall high symptom levels; and a 'Low-rising' group with mothers starting with a low symptom level that increased over time. The mothers in the High-chronic symptom group differed from the other mothers on several socio-demographic variables. They were significantly younger than the mothers in the Low group comprising the oldest mothers. The mothers in the High-chronic group had significantly lower education, were less likely to have paid work and were less likely to be living with a partner than the mothers in the other groups.

**Conclusions:**

The study shows socio-demographic differences between mothers classified into six trajectory groups based on symptoms of anxiety and depression covering 13 years of the child-rearing period. Specific socio-demographic risk factors characterised mothers in the High-chronic symptom group. Identifying subgroups with enduring problems might inform more targeted preventive efforts.

## Background

Depression and anxiety disorders are the most common mental health problems among women. The whole family is often affected and the significance of prevention and adequate treatment is high. Exploring developmental trajectories of maternal symptoms over the child-rearing period is important to improve our understanding of the variation in symptoms.

Such analytic designs require data from relatively large, population-based longitudinal studies like the current study. To our knowledge, this is the first study reporting results from examining the relationships between socio-demographic characteristics of mothers classified into trajectory groups based on symptom scores covering the child-rearing period from infancy to mid adolescence.

Depression, especially in women, is frequently found to be comorbid with anxiety [1-3]. For example, 43% of subjects in a community sample diagnosed with depression qualified for an anxiety disorder at the same time [4]. The two diagnoses might share a common genetic predisposition [4]. Nevertheless, depression and anxiety disorders are usually found to be heterogeneous conditions that vary among individuals with respect to their clinical presentations, longitudinal course, and risks of recurrence [5-7]. In this study symptoms of anxiety and depression are measured by the Hopkins Symptom Check List-25 (HSCL-25 scale) [8,9]. The scale consists of two highly correlated symptom subscales of anxiety and depression and consequently the HSCL-25 scale is mostly used as a measure of 'emotional disorder' or 'emotional distress' comparable with studies of depression and/or anxiety.

Depression is usually found to be particularly prevalent in the life phase when women give birth and raise children [10-12], but there are conflicting results as to the developmental pathways. Some studies conclude that the prevalence of symptoms of depression amongst mothers are quite stable from childbirth and onwards [6,13,14]. Others have found the risk for depression to increase in the post partum period [15] and even to increase further as the child grows older [16]. In contrast, others claim that the birth of a child might lead to a period with improved mental health for the mother [16]. However, there is still a lack of population-based studies of developmental trajectories following mothers throughout the whole child-rearing phase and there are few longitudinal studies focusing on both symptoms of depression and anxiety [17]. Studies of trajectories of psychopathology are especially useful for the investigation of transitional points in development across the life phases. Development extends throughout the entire course of life influenced by adaptive and maladaptive processes [18]. Person-centred approaches can help elucidate why the trajectories of some individuals differ from those of others. This approach is appropriate when developmental trajectories are assumed to systematically differ across individuals or when developmental changes are assumed to carry different implications for long-term individual outcomes [19,20]. This is often the case within the field of depression and anxiety disorders.

A recent review of population-based studies of trajectories of depression and anxiety disorders conclude that this research is in its infancy [7]. Nandi and colleagues found 29 studies of trajectories of which only eight were on adults, including two on mothers. They further reported that only two studies used indices based on the combination of symptoms of anxiety and depression. Colman and colleagues (2007) studied trajectories of symptoms of anxiety and depression over the life course in both men and women from the age of 13 to 53 [21], whereas Merikangas and colleagues (2003) studied clinically diagnosed anxiety and depression of both men and women [22].

The trajectory studies of mothers focused only on symptoms of depression. Campbell and colleagues classified 1,261 mothers from a population-based sample into the following six trajectory groups based on seven waves of symptom scores collected when the children were from 1 month to 7 years; High-chronic, Moderate-increasing, High-decreasing, Intermittent, Moderate-stable and Low-stable [23].

Campbell and colleagues have more recently published results from another study of the same sample using early socio-demographic risk variables and chronicity and severity of maternal depressive symptoms from infancy to age 12 years as predictors of offspring adjustment at 15 years of age. The mothers were then classified into the following five trajectories of depressive symptoms; Non-depressed, Early and decreasing, Stable subclinical, Moderately elevated, and Chronic [24]. They have not yet; however, identified trajectories based on maternal symptom scores gathered from when their children were infants until they are well into adolescence.

Another person-centred study of maternal depression in a clinical sample was conducted by Ashman and colleagues following mothers of children from infancy through 6,5 years [25]. They studied maternal depression in relation to child behaviour over seven years in a sample of 133 mother-child dyads using clinical interviews. As a result, the mothers were classified into the following three trajectory groups based on their symptom scores; Decreasing, Chronic depressed, and Stable mild depressed.

These studies provide important knowledge about different maternal symptom trajectories. However, they are based on data from child-rearing periods in the US only. Challenges mothers meet in the US might differ somewhat from those of mothers in other parts of the world. The Scandinavian countries are characterised by more gender equality, reasonable social benefits for lone mothers, long term paid parental leave, and good access to childcare [26,27], which are all important factors for parents' wellbeing. Comparisons of symptom trajectories between countries with different child-rearing conditions enlightens discussions of relationships between the effects of basic and cultural-dependent challenges on depression and anxiety disorders. Knowledge from population based studies of mothers from other countries is needed for such comparisons. Clinical studies of small groups of mothers are not able to capture the stability and change in symptoms over the child-rearing period. Population-based studies are valuable sources for informing preventive work because the majority of people suffering from mental disorders never seek professional help [7,28].

Depressive symptoms are usually found to be associated with socio-demographic variables like gender, age, employment, education, partner status and income [10,14,29,30]. However, to our knowledge no study has reported results from examining the relationships between socio-demographic characteristics of mothers classified into trajectory groups based on symptoms covering most of the child-rearing period. The relationship between socio-demographic variables and trajectories of symptoms of depression and anxiety needs to be explored further [31] because subgroups may be exposed to specific risk factors. It is also important to know more about symptom development throughout the child-rearing period to reveal potential vulnerable phases in order to make more targeted efforts.

The aim of the study is to identify symptom trajectories of anxiety and depression describing a population-based sample of Norwegian mothers from when their children were 18 months until they were 14.5 years old. The study will also look at the relationship between socio-demographic variables characterizing the mothers classified into the different symptom trajectory groups.

## Methods

### Sample

The current study used data from the Tracking opportunities and problems - from childhood through adolescence (TOPP) study. A prospective, longitudinal study of nearly 1000 families in Norway designed to investigate the influences of environmental risk and protective factors on symptoms of mental health problems and competence among children and their parents.

The study is approved by The National Committee for Medical and Health Research Ethics (NEM) in Norway. All families from 19 geographical health care areas in eastern Norway who visited a child health clinic in 1993 for a scheduled 18 month visit were invited to complete a questionnaire (t1). More than 95% of all families in Norway with children attend the public health program eight to twelve times during the first four years of the child's life.

Of the 1081 eligible families, 913 mothers filled out questionnaires at time 1. 777 mothers (85% of t1) participated at t2 when the child was 2.5 years old, 727 (80%) at t3 (4.5 years), 505 (55%) at t4 (8.5 years), 587 (64%) at t5 (12.5 years), and 474 mothers (52%) at t6 (14.5 years). 38 mothers entered the study after time 1. Hence 951 different mothers were included at one wave or more.

### Attrition

More than 95% of the families were ethnic Norwegians, i.e. non-immigrants. Background information on the mothers who declined to respond at t1 was recorded at the child health clinic. Non-respondents did not differ significantly from respondents with respect to maternal age, education, employment status, number of children and marital status. The attrition rate is increasing with time as in most longitudinal studies. This study followed mothers from when the index child was 18 months (t1) until they were 14.5 years old (t6). The questionnaires were administered at the health care clinics for the first three time points and by post from time four; this partly explains the substantial decline from 80% to 55% in participation from t3 to t4. In our study we found that there were only small and non-significant differences between the mothers who filled in questionnaires at all waves and the mothers who only participated in the first wave with respect to age, education, number of children, financial status, social support, chronic stress, negative life events and maternal mental health symptoms scores. The only significant (p < .05) predictors of drop out were maternal education and workforce participation. Mothers who participated throughout the whole period had higher education and were more likely to have paid work.

## Measures

*HSCL*: Maternal symptoms of anxiety and depression were measured by a version of the Hopkins Symptom Check List (HSCL-25) [8,9]. An index of maternal symptoms of anxiety and depression was computed by dividing the sum of all items by the number of items. The HSCL version we used had 23 items at t1 and t2, 24 items at t3, t4 and t6, and 10 items at t5. Two of the 25 items in the HSCL scale were excluded after a pilot study in the beginning of the TOPP study because some mothers perceived the items: "thoughts of killing yourself" and "loss of sexual interest or pleasure" as offensive. From t3 the item "thoughts of killing yourself" was included in the questionnaire and the scale has been administered with 24 items in the TOPP study since then, with the exception of the fifth wave when it was reduced to 10 items. A study of comparisons of short versions of the Symptom Checklist found that all short versions showed almost equally high internal consistency, sensitivity and specificity [32]. Table 1 gives descriptive and reliability scores of the HSCL scores at all waves.

**Table 1:** Characteristics of maternal symptoms of anxiety and depression (HSCL) scores at six time points.

Time point	Index child's age	Number of items	Mean (SD)	Cronbach's Alpha	Percentage above clinical cut-off (1.75)
T1	18 months	23	1.35 (.34)	.90	12
T2	2.5 years	23	1.30 (.29)	.89	7
T3	4.5 years	24	1.28 (.29)	.90	8
T4	8.5 years	24	1.30 (.32)	.91	9
T5	12.5 years	10	1.41 (.41)	.87	14
T6	14.5 years	24	1.36 (.32)	.90	13

The HSCL-25 scale is widely used for detecting psychological problems in non-psychiatric settings. The reliability and validity of the scale has been well established [32]. HSCL-25 consists of 25 items separated into two highly correlated scales for anxiety (10 items) and depression (15 items) rated on a four-point scale ranging from 1 "not at all" to 4 "extremely" affected. The HSCL-25 scale measures symptoms and does not diagnose but it is common to use a cut-off score of 1.75 as an indication of symptoms equivalent with an anxiety or depressive disorder. A Norwegian study found that using HSCL-25 with this cut-off gave the same prevalence estimates of an anxiety and depressive disorder as a diagnostic, clinical interview (CIDI) [33,34].

The correlation between HSCL scores was significant at the .01 level from one time to the next (.67 from t1 to t2, .66 from t2 to t3, .57 from t3 to t4, .52 from t4 to t5, and .62 from t5 to t6) and over the whole period. (.36 from t1-t6).

### Socio-demographic measures

*Maternal age: *was measured at time 1 when the index child was 18 months with a mean of 30 years ranging from 19 to 46 years of age.

*Maternal education: *at t1 was measured on a scale from 1 (7 year/primary school, 10% of the sample) to 5 (more than 4 years at university or university college, 16%). The sample mean was 3 (high school) at time 1 when the index child was 18 months. Maternal education was only included at t1 because it is relatively stable in this sample. The correlations between maternal education were .93 from t1 to t2, .95 from t2 to t3, .90 from t3 to t4, .94 from t4 to t5, and .94 from t5 to t6. From t1-t6 the correlation was .80. All were significant at the .01 level.

*Workforce participation: *we coded workforce participation from the questionnaires at all six time points as whether the mothers were working full time, part time or not participating in paid work. We made a dichotomous variable where we combined working full time and part time into one category of having paid work. At t1 when the index child was 18 months 63% of the mothers worked outside the home. At time 6, when the index child was 14.5, this proportion had increased to 87%.

*Childs gender: *we recorded the index child's gender and used it in the analysis. At t1 51% were girls.

*Cohabitation status/living with a partner: *cohabitation status was measured at all six time points and was coded as whether the mothers were living with or without a partner regardless of whether they were married or not. In the Scandinavian countries cohabitation is largely indistinguishable from marriage [35]. A large study in Norway found no difference in symptoms of anxiety and depression among cohabitants than among the married [36] and this was replicated in the current study. Mothers were classified as living without a partner if they either were living alone with the child/children or together with parents or friends and the child/children.

### Statistical analyses

This study used latent profile analysis (LPA) to identify distinct trajectories of symptoms of maternal anxiety and depression. LPA is a person-centered approach which is designed to divide the population under study into a set of latent subpopulations with similar developmental pathways [37]. We used Mplus, version 5, to compute different trajectories of maternal symptoms of anxiety and depression using HSCL sum scores. We estimated models with 2 to 7 classes (later referred to as groups) and focused on the model that minimized the Bayesian Information Criteria (BIC) index as the "best" model [38]. To avoid problems with local solutions each model was estimated using 500 different sets of starting values, where each set was randomly perturbed from Mplus default starting values [39]. To minimize problems with singularities, within class time specific residual variance was constrained to be equal across time but classes were allowed to have different levels of time specific residual variance [39]. After model estimation, we used estimated posterior class membership probabilities to assign individuals to pseudo-class according to the maximum probability rule where individuals are assigned to the class for which they have the highest probability of membership. Observed pseudo-class trajectories were plotted against the model fitted class trajectories as a check on the adequacy of any solution. Due to the skewness of the HSCL scores we performed two sets of analyses; one on raw scores and one on Rasch transformed scores. Both procedures gave largely similar results.

When comparing mothers in the different symptom groups we used pseudo-classes as observed groups in further analysis instead of the latent group variable. This means that individuals are assigned to the class for which they have the highest probability of membership; hence the uncertainty of latent class membership is not taken into consideration. Even though the average posterior class probabilities for trajectory membership for our six group solution ranged from .80 to .91 there are always some ambiguities regarding group membership of some individuals. In complicated statistical procedures it is possible to include predictors in the model and hence take into account the uncertainty of latent class membership and the uncertainty associated with fitting all parts of the model, the standard errors will then be more realistic. In this study we chose a simpler approach and used pseudo-class membership and tested the groups on other variables with conventional statistical methods. To protect against the potential inflation of significance we chose a more stringent p-level than .05.

The LPA analyses were done using subjects with partial data with the assumption that the missing data was missing at random (MAR). MAR is a technical term that is often misunderstood. It means that 'missingness' does not depend on unobserved variables that have not been included in the model. The MAR assumption is not testable unless the missing data can somehow be recovered and it is not invalidated if observed variables included in the model are predictive of missingness. (See Graham, 2009, for more information on modern missing data methodology [40]).

To test for group differences we performed one-way ANOVA's in SPSS version 14.0 and used the pseudo-class variable, i.e. the mothers are assigned to the class to which they have the highest probability of membership, as the factor. Because the sample size varied in the six groups, Games-Howell tests were used as post hoc test because it does not assume equal sample size or equal population variance (we also tested with Tukey and it produced almost identical results). We conducted Chi-square tests in cross-tabulations of trajectories and the dichotomous socio-demographic variables. In addition we conducted exact analyses of single cells in the cross-tabulations based on the Fisher four-field hypergeometric distribution test with the Exacon module in Sleipner version 2.1 [41]. In Exacon, the procedure is fitting a model of independence and then looking at cells that depart significantly from independence.

The Exacon module was used to analyse if mothers in the different symptom groups scored significantly more often than expected on dichotomous socio-demographic variables. This analysis produces an exact test of single cells in a contingency table. Scores observed significantly more often than expected are referred to as types (observed > expected) and those observed less frequently than expected are referred to as antitypes (observed < expected). A stringent p level of .01 was chosen for the analysis of statistical types and antitypes as correction for the number of comparisons in each cross-table was not applied.

## Results

Trying several LPA analyses with different constraints produced very similar results. When we allowed for more than six trajectories, the low symptom trajectories were divided into more low groups. We thus got corresponding overall patterns with all the different analytic approaches. We used the raw HSCL symptom scores in the LPA and chose a solution of six trajectories based on model fit estimations (BIC). Model fit indexes (BIC (Entropy)) from two to seven classes were; 49.94 (.832), -662.40 (.793), -982.65 (.803), -1125.37 (.777), -1138.34 (.769), -1133.16 (.743), respectively.

**Figure 1:**
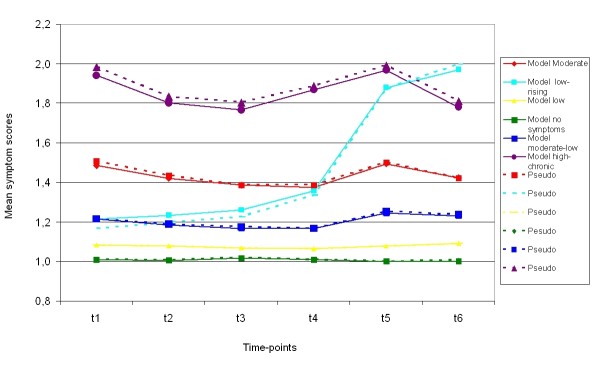
**The trajectory model of maternal symptom levels**. The coloured lines represents the six different trajectories of symptoms of anxiety and depression at six times points identified with LPA analysis. The dashed lines are the actual data (pseudo trajectories), and the solid lines are the estimated model trajectories. Maternal symptom scores are on the y-axis and data wave from t1 to t6 is on the x-axis. On the right side are names of the trajectory groups.

Figure 1 shows fitted mean trajectories from the 6 class model. It shows one trajectory without any symptoms (No symptoms, 5% of mothers at t1), one trajectory with mothers reporting few symptoms (Low, 19% of the mothers at t1), one with moderately low symptoms (Moderate-low, 30% of t1), one trajectory characterising mothers with moderate symptoms over the time span (Moderate, 32% of t1), one trajectory classifying mothers with overall high scores (High-chronic, 10% of t1) and one trajectory classifying mothers starting with low scores that increased over time (Low-rising, 4% of t1). Around half (54%) of the mothers are included in the three low symptom groups (No symptoms, Low, and Moderate-low) with stable low symptom scores over all six time points. The largest single group is the group reporting moderate symptoms with 32% of t1. 10% of the mothers are classified into the High-chronic group. This group also had the largest variation in scores, i.e. they fluctuate around the group mean level. The only group that did not have a stable symptom level over time was the Low-rising group (4%). Mothers classified into this group started with few symptoms at the first three time points (when the child was 1.5 to 4.5 years), had a moderate symptom level at t4 (8.5 years), and were above the established clinical cut-off score at t5 and t6 (when the child was 12.5 and 14.5).

### Socio-demographic variables

We further explored whether there are significant differences between the mothers in the six groups on the following socio-demographic variables: maternal age, education level, and participation in paid work, partner status and index child's gender. Table 2 and 3 present descriptive data of the continuous and dichotomous variables, respectively, and whether there are overall significant differences between the mean levels of scores among mothers classified into each of the trajectory groups.

**Table 2:** One way ANOVA analyses of maternal age and education at t1. Games Howell Post Hoc Tests of continuous variables.

	Sample mean (S.D.) n = 913	No symptoms (S.D.) n = 44	Low (S.D.) n = 175	Moderate-Low (S.D.) n = 274	Moderate (S.D.) n = 290	Low-rising (S.D.) n = 35	High-Chronic (S.D.) n = 95	F(df)	p
Maternal Age t1	30.05 (4.73)	30.85 (4.42)	30.92^a^ (4.75)	29.92 (4.82)	29.84 (4.44)	30.74 (5.02)	28.76 ^a^ (5.00)	3.343 (5)	.005
Education at t1	3.08 (1.23)	3.38 (1.11)	3.20 (1.22)	3.19 (1.20)	2.98 (1.20)	3.41 (1.26)	2.54^b^ (1.31)	5.948 (5)	.000

^a ^= significant difference between the Low and High-Chronic group (p = .007).

^b ^= the High-Chronic group is significantly different from all the other classes.

**Table 3:** Relationships between trajectory groups and workforce participation, cohabitation status and child gender at 6 time points (dichotomous variables).

	Wave	Sample mean n = 913	No symptoms n = 44	Low n = 175	Moderate-Low n = 274	Moderate n = 290	Low-rising n = 35	High-chronic n = 95	χ^2^(df)	p
Paid work	t1	63	69	67	67	62	65	44^d^	19.3 (5)	.002
	t2	63	63	58	67	63	67	58	4.3 (5)	.504
	t3	67	69	62	72	69	63	54	10.6 (5)	.060
	t4	81	90	81	83	83	91	68^c^	9.3 (5)	.099
	t5	86	87	86	90	89	87	73^c^	11.5 (5)	.043
	t6	87	94	90	91	85	86	76	9.1 (5)	.106
Living with a partner	t1	92	91	94	95	91	97	82^d^	19.3 (5)	.002
	t2	91	92	91	94	91	93	78^d^	17.4 (5)	.004
	t3	89	91	91	89	90	96	74^d^	18.0 (5)	.003
	t4	87	84	95^e^	89	87	82	71^d^	18.7 (5)	.002
	t5	84	91	90	89	81	87	65^d^	27.0 (5)	.000
	t6	83	88	91^e^	84	82	77	65^d^	15.2 (5)	.010
Index child: boys	t1	49	46	46	55^e^	48	44	41	8.7 (5)	.122

Exact analyses of single cells based on the Fisher four-field hypergeometric distribution test with the Exacon procedure:

Antitypes (observed < expected)

^c ^= observed values lower than expected, p < .01

^d ^= observed values lower than expected, p < .001

Types (observed > expected)

^e ^= observed values higher than expected, p < .01

^f ^= observed values higher than expected, p < .001

#### Age

ANOVA analysis showed an overall group difference between the mean ages of the mothers. A Games-Howell post hoc test showed that the mothers in the Low group were significantly older than the mothers in the High-chronic group. Despite the attrition this difference was significant at all time points.

#### Maternal education

ANOVA analysis of maternal education level at t1 showed that that there were overall significant differences in education level between the mothers in the six symptom trajectories. A post-hoc test showed that the mothers in the High-chronic group had a significantly lower level of education than all the other mothers. Maternal education level was relatively stable in our sample from t1 to t6 and therefore only t1 was included.

#### Workforce participation

Chi-Square tests showed that there were significant differences between the groups at t1 regarding workforce participation. The mothers in the High-chronic group were less likely to have paid work. More than half of the mothers in the High-chronic group at t1 (56%) reported not having paid work versus 31-38% of the mothers in the other groups. Analyses of single cells in the cross-tabulations showed that the proportion of mothers in the High-chronic group having paid work at t1, t4 and t5 was lower than statistically expected.

#### Cohabitation status/living together with a partner

The mothers in the High-chronic group were less likely to report living with a partner. Chi-Square tests showed that there were overall significant differences between the groups in relation to partner status at t1 when the index child was 18 months. 82% of the mothers in the High-chronic group were living with a partner at t1, compared to between 91-97% in the other groups (sample mean was 92%). This trend was stable throughout the study and we found overall significant differences in partner status between the groups at all six time points. Analyses of single cells in the cross-tabulations confirmed that a lower proportion of the mothers in the High-chronic group are living with a partner than statistically expected at all six time points. In the Low group there are more mothers reporting to live with a partner than expected at t4 and t6. The percentages in the category not living together with a partner was steadily increasing from t1 to t6 both in the sample as a whole (sample mean from 8% to 17%) and for all the groups separately. Analyses of single cells in the cross-tabulations showed that a larger proportion of mothers in the High-chronic group were living without a partner than expected at all time points. In the Low group fewer than expected were living without a partner at t4 and t6.

#### Gender of the index child

The proportion of boys and girls in the sample was similar to the general population; 51% girls and 49% boys. The range in the groups is 41-55%. Analyses of single cells in the cross-tabulations showed that the proportion of boys is higher than expected in the Moderate-low group.

## Discussion

Six trajectories of symptoms of anxiety and depression were identified in a longitudinal population-based sample of mothers in Norway with latent profile analysis (LPA). We found a No symptom group, a Low, a Moderate-low, a Moderate, a High-chronic, and a Low-rising symptom group. Although 95% of the mothers have some symptoms at one time point or another, only 10% had increased symptom levels throughout the whole child-rearing period and 32% were classified into the trajectory group with a moderate symptom level. The largest differences in socio-demographic characteristics were found between mothers in the High-chronic group and the other mothers. The mothers in the High-chronic group were significantly younger than the mothers in the Low group and they had significantly lower education than all the other mothers. The mothers in the High-chronic group were also less likely to be living with a partner and less likely to have paid work than the mothers in the other groups. In the following section we will discuss our main findings.

### Trajectories

The best fitting model gave six trajectories including proportions of mothers varying from 4% to 32%. One trajectory classifying mothers without symptoms (No symptoms, 5% of t1), one including mothers reporting few symptoms (Low, 19% of t1), one pertaining to mothers with moderately low symptoms (Moderate-low, 30% of t1), one characterising mothers with moderate symptoms (Moderate, 32% of t1), one classifying mothers with overall high scores (High-chronic, 10% of t1) and, one trajectory classifying mothers starting with low scores that increased over time to a level comparable to a clinical level (Low-rising, 4% of t1). Inspection of the raw data showed more symptoms to be reported at t5 than on other time points. To test if this was due to the different amount of items at each time point we selected the corresponding 10 items at all six time points and compared the mean values on the new summative indexes. The mean was still highest at t5; hence the mothers did report the most symptoms at time 5 when the index child was 12.5 years old. All in all, the result indicates that the majority of the mothers do endorse some of the items of the scale during the study. Only 5% of the mothers' do not report any symptoms at all six time points. Around half of the mothers are in the three trajectory groups characterized by low symptom scores. Considering that this is a longitudinal population-based sample we expected most of the mothers to be low in symptoms. In this regard it is interesting that the Moderate group is the largest single group with 32% of the mothers. This indicates that in a population-based sample there is a substantial proportion of mothers reporting mild symptoms. The High-chronic symptom group consists of 10% of the mothers which is equivalent to findings from other population-based samples.

There are few other population-based and person-oriented studies on maternal mental health. Campbell and colleagues [23,24] have published results from two interesting person-centred studies using data from the NICHD study identifying maternal depressive symptom trajectories. They have not, however, identified trajectories using data describing the whole child-rearing period, from the early preschool years to well into adolescence. Other studies of trajectories of symptoms of depression and/or anxiety have focused on clinically depressed mothers only [25], special samples like adolescent mothers [42], or on adults in general, not mothers [21].

There are also interesting similarities between these studies and the current study despite differences in sample and design. Compared to our trajectory groups, the decreasing group Ashman and colleagues indentified in their data is of specific interest [25]. They found a three-group solution with one decreasing group, one chronic depressed group and one stable mild depressed group. Their decreasing group had high level of symptoms during the child's first year of life, decreasing to a moderate level during the child's preschool years and increased again during the child's school years. This group corresponds to our Low-rising group in that we see an increase in symptoms from around the time the index child starts school. We do not have information about the symptom level of the mothers in the TOPP study during pregnancy and the first 18 months of the index child's life so we do not know whether the mothers in the Low-rising group were high or low in symptoms during their pregnancy and post partum.

One reason for the discrepancy between our findings and findings from the two other studies of maternal trajectories might be due to differences in measurement instruments and samples. Unlike the other maternal trajectory studies we used a measure combining symptoms of anxiety and depression. Our data covered a time span of 13 years. Colman and colleagues (2007) studied trajectories of symptoms of anxiety and depression over the life course in both men and women from the age of 13 to 53 [21]. They identified six trajectories of symptoms of anxiety and depression; absence of symptoms, repeated moderate symptoms, adult-onset moderate symptoms, adolescent symptoms with good adult outcome, adult-onset severe symptoms, and repeated severe symptoms. Those with symptoms, especially in adulthood, were more likely to be women. Taking into consideration the trajectories in adulthood the trajectory pattern is quite similar to ours and for example the adult-onset severe symptoms group might be the equivalent to our low rising group.

Campbell and colleagues identified six trajectories of maternal depressive symptoms in their study from 2007 [23]. The number of trajectories was similar to ours, but the group pattern was somewhat different. Like us, they report a chronic high group, a low symptom group and a moderate symptom group. In addition, they identified an intermittent group, a moderate increasing group, and a high decreasing. We identified two low groups and a low increasing group. The maternal trajectories they identified were based on data collected when the index child was from 1 month until 7 years. We continued collecting data until the child was 14.5 years old. This covers a time span more similar to a later study from Campbell and colleagues where they followed the mothers from when the child was 1 month until they turned 12 years of age [23,24]. They identified the following five trajectory groups: non-depressed, early and decreasing, stable subclinical, moderately elevated, and chronic. Again the trajectory pattern is partly different from ours in that we did not identify the early and decreasing group. This might be because our study covers a different time period. The differences are not great - we started when the children were one year older and continued until they were 2 1/2 years older - but both infancy and early adolescence are likely to have substantial impact on the mothers' symptom level. The early and decreasing group identified by Campbell and colleagues had a high symptom level during the first 15 months of the index child's life, a time when we do not have data. The other groups from Campbell and colleagues' study in 2009 corresponded well with ours except for the Low-rising group that we identified [23,24]. Campbell and colleagues identified a moderate increasing group which might be the closest to our Low-rising group. The difference is that the mothers in our Low-rising group reported a lower symptom level before the children were at the age of 4, and a steeper increase after the children reached that age.

### Socio-demographic variables

It was mainly the mothers from the High-chronic group that differed from the rest. The mean age of the mothers in our High-chronic group was lower than in all the other groups. They were significantly younger than the mothers in the Low group comprising the oldest mothers. The mothers in the High-chronic group were also less likely to be living with a partner than the mothers in the other groups. A smaller proportion of them were living with a partner than statistically expected at all six time points. The mothers in the High-chronic group also had significantly lower education than all the other mothers. It is worth noticing that the mothers in the Low-rising group had the highest education. In addition, the High-chronic mothers were less likely to have paid work. The amount of mothers having paid work increased steadily in the sample as a whole. When the index child was 18 months 64% of the mothers had paid work compared to near 88% when the child was 14.5 years old. It is more common for mothers to join the workforce as the child grows older. Contrary to this we found a decrease in workforce participation among the mothers in the Low-rising group (with high education) parallel with the increase in symptoms of anxiety and depression. This decrease seems to be parallel and to follow after the increase in symptoms. It seems that the direction was from increase in symptoms to drop out of workforce and not from being unemployed leading to increase in symptoms. It is, however, necessary to examine this further in a full model controlling for relevant mediators.

Our findings on socio-demographic variables are in line with results reported from both studies by Susan Campbell and colleagues. In their study published in 2009 they found that women with few symptoms were more likely to be married, better educated, and in better physical health than women with more elevated symptoms. In their study from 2007, Campbell and colleagues found that women in the different trajectory groups varied widely in co-occurring socio-demographic risk. Mothers in the low-stable depression trajectory group were older, better educated, and had higher incomes than the mothers in the other five trajectory groups. Women in the moderate-stable depression group were also somewhat better-off financially and had higher educational levels than women in the chronic and high-decreasing trajectories. Women in the low-stable depression group were also more likely to be in a stable marriage [23].

On the contrary, Ashman and colleagues did not find significant differences between the mothers in their study on any socio-demographic variables like mother's ethnicity, partner status, education, occupation level, or hours spent working outside the home. The lack of differences between their groups and ours might partly be because the sample consisted of depressed mothers only in contrast to our study comprising a population-based sample [25].

In our study we found the level of symptoms measured at the six time points to be quite stable over time among mothers classified into all trajectory groups except for the Low-rising group. The Low-rising group was a small group, only 4% of our sample. However, the group had low attrition; the analysis required the mothers to be part of most data waves to be included in this particular group. It is interesting to note that this is a group with a high proportion of highly educated mothers. In the Low-rising group 24% of the mothers had more than four years of university or university college education. This was only the case for 13% of the mothers in the High-chronic group. The range was from 14-17% for the mothers in the other groups. All in all, this study found that although some variables like workforce participation and partner status changed for the mothers in the Low-rising group, this group of mothers did not differ significantly from the others on most variables included. The substantial increase in reported symptom level for this particular group of mothers can not be explained by variables included in the current study.

Our study shows that for some mothers the symptoms of anxiety and depression can be a stable problem over a substantial period of time. The results in Tables 2 and 3 imply that t1 demographics significantly discriminate the groups. The high symptom mothers were associated with specific risk factors like young age, low education, living without a partner and not having paid work. The results for maternal education specifically indicate that the high chronic group is significantly different from the other groups but the other groups do not differ among themselves. Hence it is important to pay attention to specific risk factors among mothers in order to establish effective intervention and prevention programs. Perhaps even more important, education and work force participation are malleable risk factors that can be changed through effective intervention.

#### Strengths and weaknesses

There are several statistical approaches that can be used when studying person oriented trajectories. In this study a latent profile analysis (LPA) was used to identify trajectories of symptoms of anxiety and depression. The six groups based on maternal symptom scores gave a meaningful result, but there are also some limitations. For simplicity, we used pseudo-classes as observed groups in analysis of socio-demographic variables instead of predicting latent classes directly in the LPA model. This means that the uncertainty of latent class membership was not taken into consideration. To protect against the potential inflation of significance that comes when pseudo-class is treated as observed class a more stringent p level than .05 was applied.

The data in this study stems from a population-based sample which reduces the selection bias associated with studies of maternal depression and/or anxiety using clinical samples. Few studies of mental health have followed mother-child dyads over such a long time span as we have in this study. Despite the attrition that ordinarily is connected to the length of the study we still find that the remaining sample shows few differences on our study variables compared to the drop outs. Attrition analysis of the current study found that the only significant predictors of drop out were maternal education and workforce participation. Mothers who participated throughout the whole study period had higher education and were more likely to have paid work. In addition, modern statistical methods that assume that data are missing at random conditional on predictors included in the model (MAR assumption) and thus include subjects with partial data that we used tend to minimize attrition bias. We did not, however, include the variables that predicted attrition in the LPA analyses so this remains a potential limitation of our results.

The results would be strengthened if we had collected data through clinical diagnostic interviews of mothers instead of self reported symptoms on a symptom check-list. However, when the purpose is to gain knowledge that can be generalised to the population, self-reported symptom measures are found to be a valid indicator in population-based studies [33,43].

This study showed that the onset of serious symptoms of depression and anxiety might appear later in the child-rearing phase, hence it is not only the post partum time that might be a vulnerable period for mothers. It is necessary to explore the symptom trajectories further to see whether classification into one of the groups is predicted by different factors, and whether risk and protective factors might affect groups of mothers differently at different times, i.e. to identify vulnerable periods and resilient outcomes.

## Conclusions

Even though extensive research has been done on maternal depression and anxiety disorders, there are few longitudinal studies following the symptom level among mothers from the ordinary population over the whole child-rearing period. This is one of few studies that have examined the relationship between socio-demographic characteristics of mothers classified into trajectory groups based on symptom scores covering the period from when they had infants until the children were well into adolescence. It is interesting that the risk factors are consistent for the high symptom group throughout the child-rearing phase, not only at one time point. This study shows that the course of symptoms of anxiety and depression is heterogeneous and that it is useful to apply person-centred analyses in order to develop preventive initiatives. In future studies it will be important to test hypotheses about early predictors that can identify high risk group membership. Such findings might have substantial implications for early intervention and prevention of mental health problems.

## Competing interests

The authors declare that they have no competing interests.

## Authors' contributions

HJ, MS and AS performed the LPA analyses. AS performed the other statistical analyses and drafted the manuscript. KSM designed the project and collected the data. All authors contributed to the interpretation of results and helped to draft or critically revise the manuscript. All authors read and approved the final manuscript.

## Pre-publication history

The pre-publication history for this paper can be accessed here:

http://www.biomedcentral.com/1471-2458/10/589/prepub
